# Individual variation and interactions explain food web responses to global warming

**DOI:** 10.1098/rstb.2019.0449

**Published:** 2020-11-02

**Authors:** Anna Gårdmark, Magnus Huss

**Affiliations:** Swedish University of Agricultural Sciences, Department of Aquatic Resources, Skolgatan 6, SE-742 42 Öregrund, Sweden

**Keywords:** body growth, population size structure, trait variation, predator–prey, fish production, climate change

## Abstract

Understanding food web responses to global warming, and their consequences for conservation and management, requires knowledge on how responses vary both among and within species. Warming can reduce both species richness and biomass production. However, warming responses observed at different levels of biological organization may seem contradictory. For example, higher temperatures commonly lead to faster individual body growth but can decrease biomass production of fishes. Here we show that the key to resolve this contradiction is intraspecific variation, because (i) community dynamics emerge from interactions among individuals, and (ii) ecological interactions, physiological processes and warming effects often vary over life history. By combining insights from temperature-dependent dynamic models of simple food webs, observations over large temperature gradients and findings from short-term mesocosm and multi-decadal whole-ecosystem warming experiments, we resolve mechanisms by which warming waters can affect food webs via individual-level responses and review their empirical support. We identify a need for warming experiments on food webs manipulating population size structures to test these mechanisms. We stress that within-species variation in both body size, temperature responses and ecological interactions are key for accurate predictions and appropriate conservation efforts for fish production and food web function under a warming climate.

This article is part of the theme issue ‘Integrative research perspectives on marine conservation'.

## 1. Introduction

Conserving aquatic biodiversity requires knowledge of how aquatic food webs respond to global warming. Warmer waters have already led to altered species composition owing to range shifts [1] and decreased species richness [2], and could impact biomass production [3,4]. At the same time, higher temperatures often lead to higher metabolic rates [5] setting the ‘pace of life' [6] and to faster growth of individuals [7]. Observations of faster body growth but lower total biomass production of populations (in e.g. g yr^−1^; [4]; but see [8]) or food webs [3] of aquatic organisms may seem counterintuitive and the mechanisms by which these responses to climate change come about are yet not fully understood.

A key to resolve these seemingly contradictory observations of responses to warming at different levels of biological organization (from individuals to food webs) is to acknowledge that population and community responses emerge from interactions among individuals. Ecological interactions and the rate of physiological processes vary among individuals within populations, often correlated to the ‘master trait' body size [9]. Accounting for the variation among individuals within species that arises owing to food-dependent body growth and development is therefore essential to understand the dynamics and structure of animal communities [10], including their responses to environmental change. Feedbacks between changes in body size distributions and community dynamics can, for example, explain the presence of alternative stable states in food chains and the lack of recovery of overexploited predators [11,12]. Ecological interactions also depend on temperature [13] because physiological processes and activity levels do, especially in ectotherms [5,14,15]. How physiological processes, and therefore body growth, scale with body size and temperature will thus determine warming impacts on individuals, as well as how these influence size-dependent feeding interactions, and resulting feedbacks on individuals. This interplay between temperature-, food- and size-dependent physiological and ecological processes at the level of individuals and how it governs population and community responses to warming has only recently been addressed [16–18].

Recent studies show that food web responses to warming do not only depend on trophic position [19] and changes in mean population rates with temperature [6]. Warming responses also depend on trophic interactions and population size structure [16–18], which are in turn, governed by temperature- and size-dependent consumption, metabolism [5,20], energy allocation, body growth [7], reproduction and mortality. Models relying on representations of how mean survival, reproduction or population growth depend on environmental variation are therefore insufficient to identify mechanisms of food web responses to warming, as these involve feedbacks via altered body growth and population size structures. That means such models cannot fully resolve any temperature effects on food webs that modify intra- or interspecific competition or size-specific predation. Even approaches accounting for that survival and reproduction depend on body size may fall short in explaining and predicting food web responses if they only address mean rates (e.g. [21]), as it is often the variation among interacting individuals (within and between populations) that mediates the feedbacks underlying the individual, species and food web responses to warming.

Studies linking individual variation to food web responses to warming are few. Available studies provide model-based predictions [16–18] or large-scale observations (of population and not food web responses; [4]) rather than empirical tests of emergent responses to warming and their causes. Here we therefore ask: how do population and food web responses to increasing temperatures emerge from responses and processes among individuals, and is there empirical support for the mechanisms and predicted responses? To address this, we combine findings from models of simple food webs containing fish and plankton populations with those from empirical studies on fishes and aquatic invertebrates, including large temperature gradient studies and warming experiments on individuals and whole ecosystems. We focus on fishes to link individual responses to emerging effects on populations and food webs, as they are key for marine conservation and management ensuring sustainable food production from the oceans.

**Figure 1.**
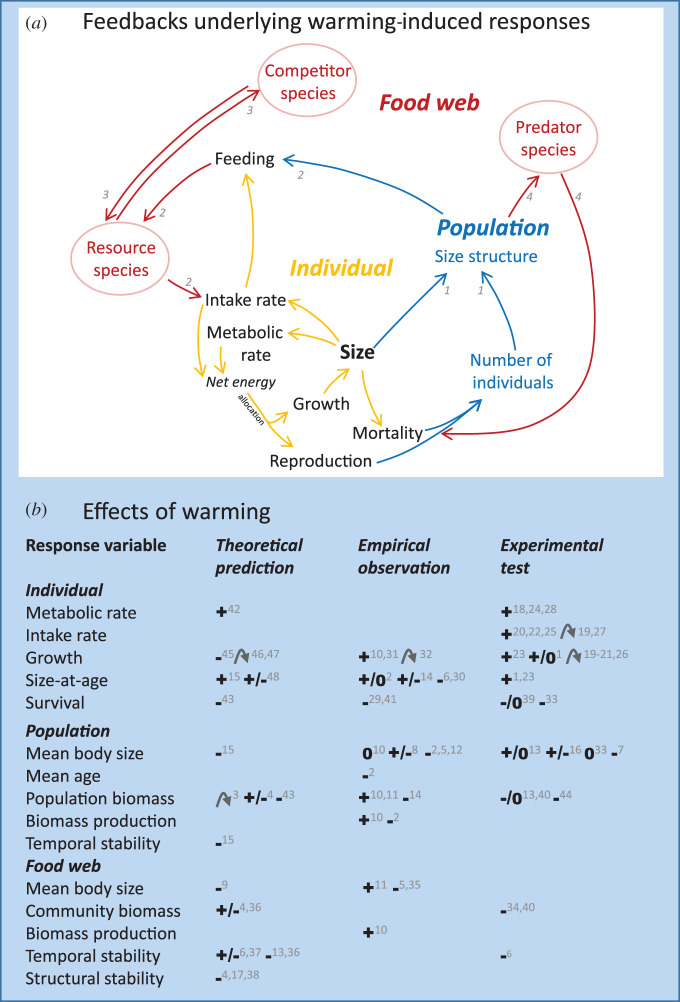
(*a*) Warming-induced responses of individuals, populations and food webs emerge from temperature-dependent rates of individual-level processes. Rates of food intake and metabolism depend on both individual body size and temperature, and size, in turn, on acquired net energy allocated to growth. Thus, warming-induced changes in these rates result in changes in the composition of populations and food webs, which feed back to affect individual growth, survival and reproduction. Such individual-level responses result from feedbacks from warming-induced shifts in population size structure (feedback indicated by *1* in (*a*)), intraspecific competition (*2*), interspecific competition (*3*) and predation (*4*). These feedbacks couple individual-level processes (yellow) to population (blue) and food web (red) dynamics, and thereby also impact how warming affects bottom-up (e.g. *3*) and top-down (e.g. *4*) regulation in food webs. (All species are size structured, but for clarity, we have illustrated this only for a focal species, and not for its predator, competitor and resource species.) (*b*) Examples of predicted, observed and experimentally tested responses to higher temperatures in aquatic systems at the levels of individuals, populations and food webs. Direction of responses is indicated by +, −, 0 or a bent arrow for hump-shaped responses. Numbers (in grey) correspond to citations listed below, where type of aquatic organism is indicated in brackets for observational and experimental studies; F = fish, Z = zooplankton, P = phytoplankton, I = insects, M = microbes, O = other: 1. [7] [F], 2. [4] [F], 3. [17], 4. [18], 5. [22] [F,Z,O], 6. [23] [M], 7. [24] [F,Z,O], 8. [25] [F], 9. [26], 10. [8] [F], 11. [27] [F,P,O], 12. [28] [F,Z,P,O], 13. [29] [M], 14. [30] [F], 15. [16], 16. [31] [ZP], 17. [32], 18. [33] [F], 19. [34] [F], 20. [35] [F], 21. [36] [F], 22. [15] [F], 23. [37] [O], 24. [38] [F], 25. [13] [I], 26. [39] [F], 27. [40] [F], 28. [41] [F], 29. [42] [F], 30. [43] [F], 31. [44] [F], 32. [45] [F], 33. [46] [I], 34. [3] [Z,P], 35. [47] [F], 36. [21], 37. [48], 38. [49], 39. [50] [I,O], 40. [51] [Z,P], 41. [52] [F], 42. [6], 43. [53], 44. [54] [P], 45. [55], 46, [56], 47. [57], 48. [58].

By linking these experimental and modelling studies we demonstrate how warming effects at different levels of biological organization (individuals, populations, food webs) are coupled (figure 1*a*). Food web responses to warming emerge from intraspecific variation in both size, interactions and temperature dependence, all ubiquitous in nature. Our review of empirical support shows that experimental tests of how warming effects on food webs arise from feedbacks via population size structures are lacking—a critical gap in our understanding of food web responses to warming.

## 2. Warming effects on individual growth

### (a) From faster feeding and metabolism to body growth

**Figure 2.**
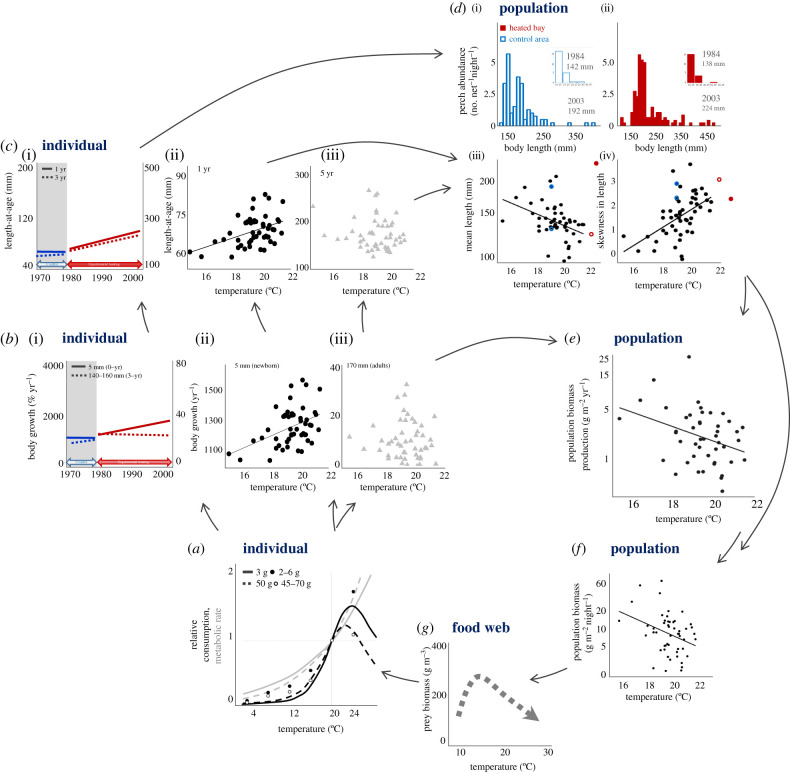
Feedbacks between size-specific individual responses to warming and population size structure and biomass. Example of warming effects on European perch (*Perca fluviatilis*), where (*a*) size-specific effects of warming on individual energy acquisition (black lines and circles) and use (grey lines) in small (full lines and filled circles) and large individuals (hatched lines and open circles) influence (*b*) body growth responses to warming, being different for small (full lines, circles) and large (hatched lines, triangles) perch individuals in the whole-ecosystem heating experiment (i) and in the lake temperature gradient study ((ii) and (iii)), and subsequently their (*c*) mean size-at-age ((i): the heating experiment, (ii) and (iii): the lake gradient). This affects (*d*) population size structure; (i,ii) show catch in numbers per unit effort per length class in the heating experiment (ii) and control area (i) and mean body size (in mm) for year 1984 and 2003 is inserted as text, in (iii, iv) black dots indicate perch in the lake temperature gradient, and coloured dots the whole-ecosystem experiment in heated (red) and control (blue) areas 4 (open circles) or 23 years (filled circles) after the onset of heating. Changes in (*e*) population biomass production over temperature result from responses in individual body growth at size (*b*) and numbers of individuals at size in the population (*d*), and lead to variation in (*f*) population biomass with temperature. The total biomass (*f*) and size composition (*d*) of individuals in the population impact their (*g*) prey at lower trophic levels (in addition to the direct influence by temperature on prey individuals). The amount of prey and its variation across temperature (*g*), in turn, influence the food intake rate of individual consumers (*a*). (i) in (*b*,*c*) are redrawn from Huss *et al*. [7], while (ii) and (iii) in (*b*,*c*), (iii) and (iv) in (*d*), and (*e,f*) are redrawn from Van Dorst *et al*. [4].

Warming-induced changes in individual body size, growth and trophic interactions are all linked (figure 1*a*) because body growth depends both on temperature, body size [53] and available energy and matter (acquired via trophic interactions). Proximately, the effects of increased temperature on body growth arise from how temperature influences energetic gains and expenditures (figures 1*a* and 2*a*) and the relative amount of energy invested in body growth versus reproduction. Underlying body growth responses to warming are thus a combination of interdependent physiological, behavioural and ecological processes (figure 1*a*). Metabolism, maintenance and tissue build-up are all physiological processes that partly depend on ecological processes (e.g. gain of energy and matter for growth depends on prey availability and predator capacity to attack and digest prey), of which all commonly depend on temperature [59]. If warming increases energetic expenditures (on e.g. maintenance) more than the processes leading up to energy gains (which often is the case; [23,60]), available net energy decreases and body growth will be slower in warm environments.

The rate of metabolism increases exponentially with temperature (figure 1*b*), as observed across animal taxa [5]. This can also speed up other physiological processes, as they are fuelled by metabolism through the oxidation of consumed carbon compounds [6]. Faster metabolism, however, requires increased energy expenditure for maintenance and repair, at least in ectotherms [61]. The amount of energy left to spend on other processes therefore depends on how maintenance costs change with metabolism, which is not known. Assuming that maintenance needs increase in direct proportion to metabolism with warmer temperatures (e.g. [6]), the energy available for growth and reproduction depends on how temperature affects food intake rates (ingestion of carbon) relative to metabolism. While energy spent on maintenance is difficult to quantify, metabolism at different temperatures can be estimated as the amount of oxygen consumed. Both maximum and standard metabolic rates in fishes are elevated at higher temperatures (e.g. [38]) but the extent is not always uniform across individuals. Standard or resting metabolic rate often increases more in large compared to small individuals ([17]; but see [20] for examples of size-independent temperature effects on metabolism). We can therefore expect variation over ontogeny in how warming affects energy expenditure, the extent depending on how metabolism and maintenance scale with temperature and body size.

Feeding rates (attack rates and/or maximum intake rates), enabling energy gain, also increase with temperature [14]. They often exhibit an optimum [14], such that consumption declines at high temperatures (figure 1*b*). How warming affects actual consumption in nature obviously also depends on available resources (and thus on trophic interactions; figure 1*a*), which often are provided ad libitum in experiments with temperature. There are no available meta-analyses of intraspecific size-dependent temperature effects on consumption as there are for metabolism [17], but examples exist where temperature effects on consumption vary within species depending on body size (e.g. European perch, *Perca fluviatilis*; [62]; figure 2*a*). Unless the temperature effects on energetic gains and costs scale identically with body size, such interactive size and temperature dependence of metabolic and intake rates means that warming will affect the net energy available for growth differently in small compared to large individuals.

### (b) Faster body growth rate of small but not large individuals

Observations indicate that the relationship between body growth and temperature differs within species depending on variation in body size among individuals [4]. This is expected if temperature affects the allometric scaling of metabolic and/or intake rates (figure 2*a,b*). This is also what controlled experiments of body growth over temperature suggest for invertebrates (e.g. red abalone *Haliotis rufescens*, [63]; the amphipod *Hyalella azteca*, [64]) and fishes (e.g. Atlantic cod *Gadus morhua*, [65], Atlantic salmon *Salmo salar*, [35]). A long-term warming experiment in which a whole (artificially enclosed) coastal ecosystem was heated also demonstrated this; individual body growth of perch was faster in the heated population compared to the control population in the surrounding archipelago with ambient temperatures—but only for small and not large individuals ([7]; figure 2*b*). This suggests that size-dependent warming effects on fish body growth are likely to occur under natural conditions.

Higher growth rates among small (and young) individuals will lead to a larger size at age among both small and large individuals, but to a lesser extent among large ones if only small individuals exhibit faster body growth ([4]; figure 2*c*). Warming could also decrease size at age among mature individuals despite faster growth as immatures, if warming decreases adult growth rates, if maturation size decreases with temperature [66] because of slower somatic growth when energy is diverted to reproduction (figure 1*a*), or if the allocation of energy to reproduction increases with temperature. Observations of variation in size at age with temperature in wild fishes are rarely reported; instead studies commonly report trends in mean size, mean adult size or maximum size [67]. Such population-level metrics vary not only with individual growth but also with mortality, and can therefore not be used to infer warming effects on body growth. The variation in body growth responses to high temperatures over ontogeny observed in fishes in natural systems [4] as well as experimentally in unfished populations over long time scales ([7], figure 2*b*) is associated with larger size at young ages ([4,7]; figure 2*c*), but no [4] or smaller [7] increases in size at older age (figure 2*c*). Responses to temperature differences occurring across generations may differ from plastic responses of individuals arising from changes in temperature- and size-dependent physiological and ecological processes, owing to evolutionary adaptation. For example, Huss *et al*. [7] found that warming also resulted in gradually faster growth of small individuals over more than 10 generations (figure 2*b*), suggesting that evolutionary adaptations to warming may need to be accounted for. Alternatively, the longer growth season or effects on resource availability caused by warming may enable small individuals to grow faster as well as large individuals to maintain growth, and hence increase in size at age owing to fast growth when young. Warming-induced changes in body size at age, such as these, feed back to affect the size-dependent rates of energy use and acquisition (figure 1*a*), as well as population size structure (figure 2*d*).

## 3. Warming shifts population size structure, productivity and dynamics

Changes in population size structure owing to warming is a direct result of how warming affects the size and numbers of individuals (figure 1*a*), via altered growth and mortality rates. Size-specific responses to warming in these rates (e.g. figure 2*b,c*) will therefore also influence how population size structures change with temperature (figure 2*d*). Growth and survival also depend both on body size and on resource availability. Any warming-induced change in population size structure will therefore affect individual growth and survival rates, as the number of individuals of different sizes controls the abundance of shared resources (figure 2*g*). How warming affects population size structure therefore depends on the feedback between size- and temperature-dependent body growth and intraspecific competition (figure 2).

### (a) Shifts to domination by small individuals

Warming has been predicted to shift population size structure towards more smaller bodied (and younger) individuals [16], because of the higher increase in metabolic demands in large compared to small individuals seen in many species [17,20], resulting in a greater competitive disadvantage of large individuals. Few empirical studies address shifts in size distributions within populations with warming, but instead commonly focus on community size spectrum responses (e.g. [27,51]). Shifts in size composition at the community level can result from concurrent changes in the composition of (differently sized) species, within-species changes among populations, as well as changes in size structure within populations. Correlations between community size structure and temperature can therefore not help elucidate temperature-dependent processes within populations. Another problem is that several studies reporting changes in fish population size structure (often as mean or maximum body sizes) rely on observations of temporal trends in commercially exploited populations (e.g. [22,67,68]). Such observational data suffer from the confounding effects of fishing mortality and temperature, which act in the same direction. Trends in populations becoming composed of more small (than large) individuals with increasing temperature over time do not necessarily suggest an effect of temperature when found in exploited species.

Variation in size distribution with temperature has been observed in perch populations that are not commercially exploited [4], with populations in warmer lakes having a higher proportion of small individuals (figure 2*d*). Correspondingly, mean body size in the populations decline with lake temperature (figure 2*d*), as also observed in other fish species across even larger temperature gradients [69]. Observations of changes in lake populations of multiple fish species across years also showed a higher proportion of small individuals in warm compared to cold years in some species (perch and common bream, *Abramis brama*), but not in others (common roach, *Rutilus rutilus*) [70]. That warming can increase the proportion of small individuals in fish populations seems to be corroborated by the whole-ecosystem warming experiment on an unexploited perch population, which showed a lower mean body size and increased skew in population size structure in the years following the onset of artificial heating (figure 2*d*, year 1984; see the electronic supplementary material for methods). Long-term responses may, however, be different, as exemplified by the larger mean body size and less skewed size structure in the heated compared to the natural ecosystem after 23 years of warming (figure 2*d*, year 2003). This could indicate evolutionary adaptations (as suggested by [7]), altered population dynamics (why differences in size structure between single years should be interpreted with caution) or increased ecosystem productivity. Increasing mean body size with warming has also been observed in several coral reef fish species that in general are unexploited, although negative correlations with the temperature trends were more common [71]. Experiments, on the other hand, of warming effects on size structure in fish populations are rare (figure 1*b*).

Temperature-dependent changes in mortality rate also determine how population size structures vary across temperature (figure 1*a*). Warming-induced increases in body growth [7] could alter size-dependent rates of mortality owing to e.g. predation (e.g. by decreasing time spent in vulnerable life stages; [72]) or starvation (affecting minimum energy reserves needed for winter survival; [73,74]). Tolerance to high temperatures (thermal tolerance) also varies with body size [75,76]. In addition, increased metabolic demands at higher temperatures should lead to higher starvation mortality if resources and intake rates do not increase accordingly [77]. Despite this multitude of ways by which warming is predicted to affect mortality (with subsequent effects on competition and body growth; figure 1*a*), empirical tests of mortality responses to warming in a food web context remain few. Warming experiments commonly address critical temperatures for body functioning on individuals (such as flip-over temperatures in fishes; [78]), but rarely how less harmful temperature increases may affect mortality indirectly via e.g. increased energy demands, lower food availability or increased predation rates (but see [52]).

Observations of lower mean age in warm temperatures [4] could be explained by higher mortality at old ages (/large sizes), corresponding to greater warming-induced increases in energy needs for large individuals [17], but could also be owing to higher birth rates [16]. In a tagging study carried out during the first year after heating in the whole-ecosystem warming experiment, Sandström *et al*. [79] found higher mortality among mature perch individuals during spawning in the heated compared to in the control area with natural temperatures. The difference in mortality between the two temperature areas was however not sustained across years, or body sizes. Interestingly, in the second year of heating they found a decrease in maturation size in the heated area and concurrent increase in mortality of the smaller mature individuals during spawning. Sandström *et al*. [79] suggested that these small individuals with a more limited energy reserve were struck harder by the higher energy demands in the warm environment as they additionally diverted more energy to gonads compared to individuals of the same size in the natural environment. This illustrates that the feedback between size-dependent body growth, resource use, energy demands and size-dependent survival also involves energy allocation to reproduction versus growth (figure 1*a*), and how this feedback governs population responses, such as mortality rate, to warming.

### (b) Less biomass production and more cyclic dynamics

The effect of warming on fish population biomass production (figure 2*e*), a key ecosystem service, is a direct consequence of how warming changes individual growth in biomass and the number of individuals per body size (as body growth is size-specific) in the population. At first sight, population biomass production could be thought to increase with temperature because individual body growth often does [59], but such a prediction ignores variation in body size, size-dependent body growth responses to temperature and warming effects on population size structure. Accordingly, despite observing an increased growth rate early in life ((ii) in figure 2*b*), Van Dorst *et al*. [4] found a decreased population biomass production with temperature across 52 lake populations of perch (figure 2*e*). The reason is that small but not large individuals grow faster in warm lakes ((ii) and (iii), respectively in figure 2*b*) and these small individuals only constitute a small part of the total population biomasses. Their faster body growth has therefore little effect on total population biomass production. In addition, the proportion and biomass of large individuals was lower in warmer lakes (higher skewness in the population length distribution, (iv) in figure 2*d*; [4]). The fewer large individuals growing in warm lakes (at similar rates as in cold lakes), which is not compensated for by the faster growth of small individuals as they only make up a small fraction of the total biomass, results in lower biomass production of populations in warmer lakes [4]. This highlights the importance of accounting for both the size dependence of individual responses, and within-population variation in body size to explain and predict responses of ecosystem functions, such as biomass production, to warming.

Fish population biomass production could also increase with temperature. In a smaller gradient study with six geothermically heated streams, O'Gorman *et al*. [8] used individual measurements of 56 trout (*Salmo trutta*) individuals from mark-recaptures to estimate total biomass production per stream. As others (e.g. [4,7]), they found higher individual growth rates during the first year of life, but in contrast found no change in mean body size and higher biomass production in warm streams [8]. They explained the observation of a higher population biomass production, despite higher energy demands in the warm environments, with trout shifting to more energetically profitable prey and an overall increase in trophic efficiency in the food webs at high temperatures. The estimate of total population biomass production was, however, derived from mean body growth rate and mean mass in the populations [8], ignoring any effects of warming-induced shifts in population size structure and size-dependent body growth. It is thus impossible to know how well the estimate approximates actual warming effects on biomass production of the studied populations, as it was derived without accounting for the variation in biomass and growth among individuals. Their study nevertheless highlights that temperature effects on lower trophic levels and feeding behaviour may also influence how consumer population biomass production changes with warming.

Size-dependent warming effects on energy intake and use do not only influence the structure and production of populations, it also results in shifts in population dynamics with warming. Models with explicit resource dynamics predict that warming will reduce cycles in consumer populations, if maintenance costs increase more with temperature than intake rates and resource carrying capacities do, because consumer density then decreases and consumer-resource cycles weaken [18,23]. Models accounting for intraspecific size variation and resulting intraspecific competition instead show that the opposite effect of warming can occur [16]. In species with size-dependent temperature effects on intake rate or metabolic rate [17,20,38], warming increases the competitive disadvantage of large individuals relative to small ones. Increasing temperatures can therefore shift the dynamics from small amplitude cycles with multiple coexisting generations to what is known as cohort cycles, where numerous small individuals of the same age outcompete their parental generation, grow to maturation and are in turn outcompeted by their young. Populations are therefore predicted to have a more even distribution of body sizes in cold compared to warm environments [16]. This corresponds to observed shifts towards a higher proportion of small individuals in fish populations with temperature [4,69,70], but studies on how warming affects the temporal dynamics of natural populations are missing. Experimental studies of temperature effects on population cycles have commonly been undertaken in microcosms [3,23,48], which may limit inferences of mechanisms for larger organisms. For example, in experiments on bacteria and their ciliate predators, Fussmann *et al*. [23] showed that warming dampened ciliate cycles. While this supports predictions from models lacking size structure in consumer populations (e.g. [23]), it cannot be used to test the mechanisms proposed by Ohlberger *et al*. [16] as there was no variation in size of ciliates or bacteria in the experiment, or a response in size to temperature [23]. To test the role of this feedback between size- and temperature-dependent rates of individuals' energy gain and use and intraspecific competition, experiments of how warming alters population size structures and resulting population dynamics are thus called for.

## 4. Emergent warming effects on communities and food webs

The environment an individual experiences consists of abiotic conditions and other individuals, of the same and other species. Shifts in the number of individuals of different sizes (i.e. in population size structure, figure 2*d*) therefore feed back on their growth (figure 2*b*) and reproduction, and hence also on individuals' trophic interactions (e.g. figure 2*g*). These feedbacks between individual performance and population size- and stage structure can determine both dynamics and structure of simple food webs (e.g. [11,80,81]) and explain their responses to management actions (e.g. [82]). Warming can therefor alter food webs directly via e.g. temperature-dependent species interactions (e.g. attack rates; [14]) varying in sensitivity to temperature among species [59], or owing to altered spatial overlap of predators and prey due to warming-driven range shifts [83]. In mesocosm experiments on larval dragonflies, for example, warming increased the rate of intraguild predation by increasing feeding rates [13]. Not only does warming alter interaction strength, but interactions determine how food webs respond to warming [18,47,84,85]. Observations in natural communities, as well as experimental evidence, show that responses to warming vary depending on food web structure [50,86]. In a mesocosm experiment with tadpole larvae and dragonflies as predators, Rudolf & Roman [50] showed that high temperatures can strongly reduce herbivore survival in single species treatments, but have no measurable effects in the presence of an inferior competitor and a predator. This exemplifies how we cannot predict effects of warming without accounting for species interactions and food web structure.

### (a) Shifts in dominant interactions

Metabolism of heterotrophs increases more with temperature than metabolism of autotrophs does, and warming is therefore generally predicted to increase consumer top-down control of resources [3], depending on the temperature sensitivity of energy gain relative to energy expenditure within species [21] and between species at different trophic levels [23] as well as on resource availability [3]. If consumers' food intake rates increase more with temperature than their metabolism does, Vasseur & McCann [21] predicted that resource biomass would decrease and consumer biomass increase, and vice versa, given that there is no direct temperature effect on the resource species. The responses depend on the temperature sensitivity of resource carrying capacity relative to that of the consumers feeding rates (half-saturation density and maximum intake) as well as metabolic rate [23]. While biomass of primary producers are often assumed (based on the metabolic theory of ecology [6]) to decline with warming, satellite data from large lakes across the globe indicate that in about half the cases, the amount of primary producers increased with lake temperature, depending on trophic state of the lake [60]. Microcosm experiments with phyto- and zooplankton populations give support to the former prediction by Vasseur & McCann [21], demonstrating a lower resource biomass despite higher productivity and a higher biomass of consumers relative to resources at higher temperature [3,32,51]. The opposite responses occurred, however, when phytoplankton populations were resource limited [3], demonstrating the importance of resource-dependent growth for food web responses to warming.

While the effect of warming on top-down control seems somewhat predictable in two-species systems with auto- and heterotrophs, the response in larger food webs depends on at which trophic level it is measured [19,32] and the type of species interactions involved. Experiments on tri-trophic food chains have found warming to increase top-down control in terms of trophic cascades from top-consumers to primary producers [19,87], but no measurable effect on top-down control of the intermediate consumers [19]. By contrast, both interspecific competition and predation decreased with temperature in mesocosm experiments with simple food webs (competitive and diamond-shaped webs; [50]). Variation in the effects of warming on top-down control has been ascribed to the prevailing temperature environment, with stronger effects in cold environments [88] or during cold seasons [89]. Rudolf & Roman [50] instead suggested that the lack of top-down control in warm environments can be explained by warming-induced changes in body growth, with faster prey growth rates in warm environments enabling them to outgrow the range of sizes where they are vulnerable to predation (figure 1*a*). Differences in temperature-driven changes in body growth, and hence in the ability to outgrow size-specific predation windows, were also suggested as the cause for different impacts of warming on the biomass of the two prey species in the experiments [50]. Models ignoring intraspecific body size variation or body growth (e.g. [21,23,77]) and their temperature and size dependence are unable to explain such observed responses to warming. Predictions of food web responses to warming ignoring these mechanisms are therefore probably overly simplistic.

### (b) Warming-induced collapse of predators

Body growth and within-species size variation can govern food web responses to warming, even in cases where temperature effects on individual net energy gain do not change with body size within species. Across species, the impacts of temperature depend on body size, such that warming-induced reductions in body size have been found to be greater in larger bodied aquatic organisms [24]. Correspondingly, species maximum body sizes can predict observed responses in body size and abundance to climatic variability in natural fish assemblages, also when accounting for exploitation by fisheries [68]. This suggests that large-bodied species, i.e. predators will be struck harder than small-bodied by warming.

**Figure 3.**
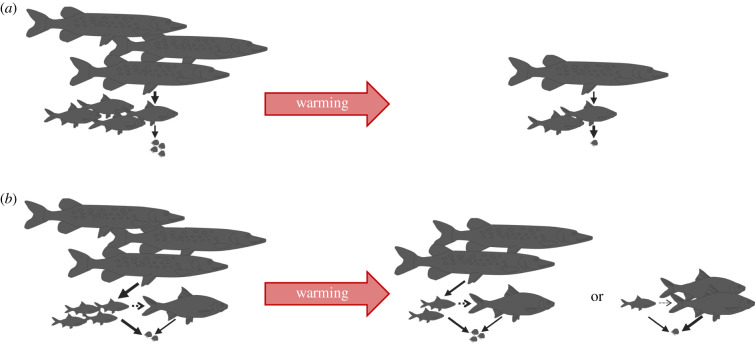
Warming impacts on predator–prey food chains depend on within-species variation (here individual variation in predation risk). Food chain models that (*a*) ignore within-species variation predict that warming will lead to gradual declines in top-predators and intermediate consumers (as well as resources, when these are also directly impacted by temperature), whereas a model that (*b*) accounts for within-species variation in size and corresponding vulnerability to predation in intermediate consumers, demonstrates that warming can lead to alternative stable states in the food chain and sudden predator collapses. In both cases, warming weakens top-down control owing to bottom-up effects of declining resources and a stronger warming-induced decrease in net energy gain of top-predators than of their prey, the intermediate consumer. However, when predation is size-dependent (*b*), weakened top-down control with warming can instead lead to a decrease in the prey that is vulnerable to predation (in *b*: small life-stage of the intermediate consumer), and collapse of top-predator populations owing to a lack of prey (right-most food web illustration in *b*). (Online version in colour.)

Predator–prey and food chain models accounting for temperature-dependent vital rates, but ignoring body growth and intraspecific size variation (e.g. [23,48]), predict that because of the stronger effect on large-bodied species, predator biomass will gradually decline with temperature until extinction (figure 3*a*). For the many predators where feeding depends on body size [90], this may not be the case. Lindmark *et al*. [18] showed that as predators feed selectively on smaller individuals in prey populations, warming can lead to alternative stable states in the food chain and sudden predator collapses (figure 3*b*). This is because warming reduces net energy gain of predators more than of prey, which reduces their relative biomass and releases prey from predation. In species where predation leads to overcompensation of vulnerable life stages of their prey (referred to as emergent Allee effects [11]), predatory release can lead to a decrease rather than an increase of vulnerable prey. Warming-induced loss of top-down control can, in such cases result in sudden predator collapses (figure 3*b*). Experiments of how size/stage variation in prey and corresponding variation in predation rates change with temperature are rare (but see [50]), and the prediction of warming-driven predator collapses have not yet been tested.

Higher temperatures may increase top-down control in systems where size differences between predator and prey are large and prey cannot outgrow the predation window, but may otherwise result in the opposite [50], through a loss of predators owing to lack of prey of vulnerable sizes. Shortening of food chains and simplifications of food webs at higher temperatures have been observed in gradients across geothermically heated stream food webs [49]. It is also suggested to have occurred in the whole-ecosystem warming experiment [7] illustrated in figure 2, inferred from analyses of perch diets and top-down effects in a predator-exclosure experiment therein [91]. Predictions from models of simple food webs [18,23,48,49], supported by these experimental studies, suggest that warming may reduce the complexity of marine food webs. Oceans are, however, open systems with connected food webs, and none of the experiments or modelling studies above account for the fact that species' distributions can change with warming [1,92]. Shifts in the spatial overlap between species with warming will together with the processes addressed herein (figure 1), contribute to the restructuring of food webs. Although warming-induced simplifications of food webs through shortening of food chains or loss of species can have many causes, we argue that accounting for three types of within-species variation—in net energy gain, in temperature effects as well as in species interactions—reveals key mechanisms explaining food web responses to warming.

## 5. Conclusion

Warming effects in aquatic food webs on e.g. species composition and productivity, that are key for ecosystem functions and services and thus for conservation efforts, emerge from feedbacks between temperature- and size-dependent rates of individual energy gain, use and survival, intraspecific competition and interspecific interactions. A key link in these feedbacks underlying observed warming responses is population size structure, because of how it arises from, as well as influences, individual body growth and survival (figures 1*a* and 2). Observation studies accounting for within-population variation in body size and temperature responses can explain seemingly contradictory patterns of variation in individual growth and population biomass production across temperature. Models and experiments acknowledging intraspecific size variation show how warming effects on food webs are governed by size-dependent interactions and have identified novel mechanisms—mediated via population size structures—of how food webs may respond to warming. To understand how climate warming affects food webs we therefore need to account for how their responses emerge from intraspecific variation in both size, interactions and temperature dependence. Our review suggests that while model-based predictions and observation studies of how aquatic systems respond to climate warming have just begun to account for these ubiquitous forms of intraspecific variation, experimental tests are largely lacking. Specifically, experiments of warming responses in interacting species with size-dependent temperature effects on food intake and/or metabolism, where (initial) population size structures are manipulated, would be useful to test the mechanisms underlying emergent responses in e.g. food web structure, mean size and production to warmer waters. We argue that advancing understanding of global warming effects on aquatic food webs through model predictions, observations and experimental tests accounting for within-species variation in size, interactions and temperature responses is essential to support management and conservation efforts to mitigate negative effects of increasingly warmer seas, lakes and oceans.

## Supplementary Material

Methods and data for figure 2

## Data accessibility

Previously published data assembled in figure 2 can be found in Van Dorst *et al*. [4] and Huss *et al*. [7], whereas previously unpublished datasets and values recalculated from Lessmark [62] can be found in the electronic supplementary material.

## References

[1] Donelson JM et al 2019 Understanding interactions between plasticity, adaptation and range shifts in response to marine environmental change. Phil. Trans. R. Soc. B 374, 20180186 (10.1098/rstb.2018.0186) 30966966 PMC6365866

[2] Gruner DS, Bracken MES, Berger SA, Eriksson BK, Gamfeldt L, Matthiessen B, Moorthi S, Sommer U, Hillebrand H 2017 Effects of experimental warming on biodiversity depend on ecosystem type and local species composition. Oikos 126, 8–17. (10.1111/oik.03688)

[3] O'Connor MI, Piehler MF, Leech DM, Anton A, Bruno JF 2009 Warming and resource availability shift food web structure and metabolism. PLoS Biol. 7**,** e1000178 (10.1371/journal.pbio.1000178) 19707271 PMC2723928

[4] Van Dorst RM, Gårdmark A, Svanbäck R, Beier U, Weyhenmeyer GA, Huss M. 2019 Warmer and browner waters decrease fish biomass production Glob. Change Biol. 25, 1395–1408. (10.1111/gcb.14551) PMC6850179 30570185

[5] Gillooly JF, Brown JH, West GB, Savage VM, Charnov EL 2001 Effects of size and temperature on metabolic rate. Science 293, 2248–2251. (10.1126/science.1061967) 11567137

[6] Brown JH, Gillooly JF, Allen AP, Savage VM, West GB 2004 Toward a metabolic theory of ecology. Ecology 85, 1771–1789. (10.1890/03-9000)

[7] Huss M, Lindmark M, van Dorst RM, Jacobson P, Gårdmark A. 2019 Large-scale experimental evidence of gradual size-dependent shifts in body size and growth of fish in response to warming Glob. Change Biol. 25, 2285–2295. (10.1111/gcb.14637) PMC6850025 30932292

[8] O'Gorman EJ et al 2016 Temperature effects on fish production across a natural thermal gradient. Glob. Change Biol. 22, 3206–3220. (10.1111/gcb.13233) PMC4991275 26936833

[9] Peters RH 1983 The ecological implications of body size. Cambridge, UK: Cambridge University Press.

[10] de Roos AM, Persson L 2013 Population and community ecology of ontogenetic development. Princeton, NJ: Princeton University Press.

[11] de Roos A. M, Persson L. 2002 Size-dependent life-history traits promote catastrophic collapses of top predators. Proc. Natl Acad. Sci. USA 99, 12 907–12 912. (10.1073/pnas.192174199) PMC130558 12237404

[12] Gårdmark A, Casini M, Huss M, Van Leeuwen A, Hjelm J, Persson L, de Roos AM 2015 Regime shifts in exploited marine food-webs: detecting mechanisms underlying alternative stable states using size-structured community dynamics theory. Phil. Trans. R. Soc. B 370, 20130262 (10.1098/rstb.2013.0262)

[13] Frances DN, McCauley SJ 2018 Warming drives higher rates of prey consumption and increases rates of intraguild predation. Oecologia 187, 585–596. (10.1007/s00442-018-4146-y) 29687229

[14] Englund G, Öhlund G, Hein CL, Diehl S 2011 Temperature dependence of the functional response. Ecol. Lett. 14, 914–921. (10.1111/j.1461-0248.2011.01661.x) 21752171

[15] Lefébure R, Larsson S, Byström P 2014 Temperature and size-dependent attack rates of the three-spined stickleback (*Gasterosteus aculeatus*); are sticklebacks in the Baltic Sea resource-limited? J. Exp. Mar. Biol. Ecol. 451, 82–90. (10.1016/j.jembe.2013.11.008)

[16] Ohlberger J, Edeline E, Vollestad LA, Stenseth NC, Claessen D 2011 Temperature-driven regime shifts in the dynamics of size-structured populations. Am. Nat. 177, 211–223. (10.1086/657925) 21460557

[17] Lindmark M, Huss M, Ohlberger J, Gårdmark A 2018 Temperature-dependent body size effects on population responses to climate warming. Ecol. Lett. 21, 181–189. (10.1111/ele.12880) 29161762

[18] Lindmark M, Ohlberger J, Huss M, Gårdmark A 2019 Size-based ecological interactions drive food web responses to climate warming. Ecol. Lett. 22, 778–786. (10.1111/ele.13235) 30816635 PMC6849876

[19] Shurin JB, Clasen JL, Greig HS, Kratina P, Thompson PL 2012 Warming shifts top-down and bottom-up control of pond food web structure and function. Phil. Trans. R. Soc. B 367, 3008–3017. (10.1098/rstb.2012.0243) 23007089 PMC3479752

[20] Ohlberger J, Mehner T, Staaks G, Hölker F 2012 Intraspecific temperature dependence of the scaling of metabolic rate with body mass in fishes and its ecological implications. Oikos 121, 245–251. (10.1111/j.1600-0706.2011.19882.x)

[21] Vasseur DA, McCann K. S 2005 A mechanistic approach for modelling temperature-dependent consumer-resource dynamics. Am. Nat. 166, 184–198. (10.1086/431285) 16032573

[22] Daufresne M, Lengfellner K, Sommer U 2009 Global warming benefits the small in aquatic ecosystems. Proc. Natl Acad. Sci. USA 106, 12 788–12 793. (10.107/pnas.0902080106) 19620720 PMC2722360

[23] Fussmann KE, Schwarsmüller F, Brose U, Jousset A, Rall BC 2014 Ecological stability in response to warming. Nat. Clim. Change 4, 206–210. (10.1038/nclimate2134)

[24] Forster J, Hirst AG, Atkinson D 2012 Warming-induced reductions in body size are greater in aquatic than terrestrial species. Proc. Natl Acad. Sci. USA 109, 19 310–19 314. (10.1073/pnas.1210460109) PMC3511100 23129645

[25] Sheridan JA, Bickford D 2011 Shrinking body size as an ecological response to climate change. Nat. Clim. Change 1, 401–406. (10.1038/nclimate1259)

[26] Cheung WWL et al 2013 Shrinking of fishes exacerbates impacts of global ocean changes on marine ecosystems. Nat. Clim. Change 3, 254–258. (10.1038/NCLIMATE1691)

[27] O'Gorman EJ et al. 2017 Unexpected changes in community size structure in a natural warming experiment. Nat. Clim. Change 7, 659–663. (10.1038/NCLIMATE3368)

[28] Gardner JL, Peters A, Kearney M, Joseph L, Heinsohn R 2011 Declining body size: a third universal response to warming? Trends Ecol. Evol. 26, 285–291. (10.1016/j.tree.2011.03.005) 21470708

[29] Tabi A, Petchey OL, Pennekamp F 2019 Warming reduces the effects of enrichment on stability and functioning across levels of organisation in an aquatic microbial ecosystem. Ecol. Lett. 22, 1061–1071. (10.1111/ele.13262) 30985066

[30] Crozier LG, Zabel RW, Hockersmith EE, Achord S 2010 Interacting effects of density and temperature on body size in multiple populations of Chinook salmon. J. Anim. Ecol. 79, 342–349. (10.1111/j.1365-2656.2009.01641.x) 20002859

[31] Sebastian P, Stibor H, Berger S, Diehl S 2012 Effects of water temperature and mixed layer depth on zooplankton body size. Mar. Biol. 159, 2431–2440. (10.1007/s00227-012-1931-8)

[32] Petchey OL, McPhearson PT, Casey TM, Morin PJ 1999 Environmental warming alters food-web structure and ecosystem function. Nature 402, 69–72. (10.1038/47023)

[33] Elliott JM 1976 The energetics of feeding, metabolism and growth of brown trout (*Salmo trutta* L.) In relation to body weight, water temperature and ration size. J. Anim. Ecol. 45, 923–948. (10.2307/3590)

[34] Koskela J, Pirhonen J, Jobling M 1997 Feed intake, growth rate and body composition of juvenile Baltic salmon exposed to different constant temperatures. Aquacult. Int. 5, 351–360. (10.1023/A:1018316224253)

[35] Handeland SO, Imsland AK, Stefansson SO 2008 The effect of temperature and fish size on growth*,* feed intake*,* food conversion efficiency and stomach evacuation rate of Atlantic salmon post*-*smolts. Aquaculture 283, 36–42. (10.1016/j.aquaculture.2008.06.042)

[36] Lefébure R, Larsson S, Byström P 2011 A temperature dependent growth model for the three-spined stickleback *Gasterosteus aculeatus*. J. Fish Biol. 79, 1815–1827. (10.1111/j.1095-8649.2011.03121.x) 22141889

[37] Hoefnagel KN, Verberk WCEP 2015 Is the temperature-size rule mediated by oxygen in aquatic ectotherms? J. Therm. Biol. 54, 56–65. (10.1016/j.jtherbio.2014.12.003) 26615727

[38] Messmer V, Pratchett MS, Hoey AS, Tobin AJ, Coker DJ, Cooke SJ, Clark TD 2017 Global warming may disproportionately affect larger adults in a predatory coral reef fish. Glob. Change Biol. 23, 2230–2240. (10.1111/gcb.13552) 27809393

[39] Elliott JM 1975 The growth rate of brown trout (*Salmo trutta* L.) fed on maximum rations. J. Anim. Ecol. 44, 805–821. (10.2307/3720)

[40] Elliott JM 1975 Number of meals in a day, maximum weight of food consumed in a day and maximum rate of feeding for brown trout, *Salmo trutta* L. Freshwat. Biol. 5, 287–303. (10.1111/j.1365-2427.1975.tb00142.x)

[41] Sandblom E et al. 2016 Physiological constraints to climate warming in fish follow principles of plastic floors and concrete ceilings. Nat. Commun. 7, 11447 (10.1038/ncomms11447) 27186890 PMC4873662

[42] Martins EG, Hinch SG, Patterson DA, Hague MJ, Cooke SJ, Miller KM, Lapointe MF, English KK, Farrell AP 2011 Effects of river temperature and climate warming on stock-specific survival of adult migrating Fraser River sockeye salmon (*Oncorhynchus nerka*). Glob. Change Biol. 17, 99–114. (10.1111/j.1365-2486.2010.02241.x)

[43] Rountrey A, Coulson PG, Meeuwig JJ, Meekan M 2014 Water temperature and fish growth: otoliths predict growth patterns of a marine fish in a changing climate. Glob. Change Biol. 20, 2450–2458. (10.1111/gcb.12617) 24862838

[44] Houde ED 1989 Comparative growth, mortality, and energetics of marine fish larvae: temperature and implied latitudinal effects. Fish Bull. US 87, 471–495.

[45] Neuheimer AB, Thresher RE, Lyle JM, Semmens JM 2011 Warming waters exceed tolerance limit for fish growth. Nat. Clim. Change 1, 110–113. (10.1038/nclimate1084)

[46] McCauley SJ, Hammond JI, Mabry KE 2018 Simulated climate change increases larval mortality, alters phenology, and affects flight morphology of a dragonfly. Ecosphere 9, e02151 (10.1002/ecs2.2151) 30555728 PMC6290685

[47] Edeline E, Lacroix G, Delire C, Poulet N, Legendre S 2013 Ecological emergence of thermal clines in body size. Glob. Change Biol. 19, 3062–3068. (10.1111/gcb.12299) 23780903

[48] Binzer A, Guill C, Brose U, Rall BC 2012 The dynamics of food chains under climate change and nutrient enrichment. Phil. Trans. R. Soc. B 367, 2935–2944. (10.1098/rstb.2012.0230) 23007081 PMC3479739

[49] O'Gorman EJ et al 2019 A simple model predicts how warming simplifies wild food webs. Nat. Clim. Change 9, 611–616. (10.1038/s41558-019-0513-x)

[50] Rudolf VHW, Roman A 2018 Trophic structure alters consequences of environmental warming. Oikos 127, 1646–1656. (10.1111/oik.05535)

[51] Yvon-Durocher G, Montoya JM, Trimmer M, Woodward G 2011 Warming alters the size spectrum and shifts the distribution of biomass in freshwater ecosystems. Glob. Change Biol. 17, 1681–1694. (10.1111/j.1365-2486.2010.02321.x)

[52] Biro PA, Post JR, Booth DJ 2007 Mechanisms for climate-induced mortality of fish populations in whole-lake experiments. Proc. Natl Acad. Sci. USA 104, 9715–9719. (10.1073/pnas.0701638104) 17535908 PMC1887605

[53] Savage VM, Gillooly JF, Brown JH, West GB, Charnov E 2004 Effects of body size and temperature on population growth. Am. Nat. 63, 429–441. (10.1086/381872) 15026978

[54] Bernhardt JR, Sunday JM, O'Connor MI 2018 Metabolic theory and the temperature-size rule explain the temperature dependence of population carrying capacity. Am. Nat. 192, 687–697. (10.1086/700114) 30444656

[55] Von Bertanlanffy L 1960 Principles and theory of growth. In Fundamental aspects of normal and malignant growth (ed. Nowinski WW), pp. 137–259. New York: NY: Elsevier.

[56] Sharpe PJ.H, DeMichele DW 1977 Reaction kinetics of poikilotherm development. J. Theor. Biol. 64, 649–670. (10.1016/0022-5193(77)90265-X) 846210

[57] Schoolfield RM, Sharpe PJH, Magnuson CE 1981 Non-linear regression of biological temperature-dependent rate models based on absolute reaction-rate theory. J. Theor. Biol. 77, 608–620. 10.1016/0022-5193(81)90246-0 6790878

[58] Atkinson D 1994 Temperature and organism size – a biological law for ectotherms? Adv. Ecol. Res. 25, 1–58. (10.1016/S0065-2504(08)60212-3)

[59] Dell AI, Pawar S, Savage VM 2011 Systematic variation in the temperature dependence of physiological and ecological traits. Proc. Natl Acad. Sci. USA 108, 10 591–10 596. (10.1073/pnas.1015178108) PMC3127911 21606358

[60] Kraemer BM, Mehner T, Adrian R 2017 Reconciling the opposing effects of warming on phytoplankton biomass in 188 large lakes. Sci. Rep. 7, 10762 (10.1038/s41598-017-11167-31) 28883487 PMC5589843

[61] MacFadyen EJ, Williamson CE, Grad G, Lowery M, Jeffrey WH, Mitchell DL 2004 Molecular response to climate change: temperature dependence of UV-induced DNA damage and repair in the freshwater crustacean *Daphnia pulicaria*. Glob. Change Biol. 10, 408–416. (10.1111/j.1529-8817.2003.00750.x)

[62] Lessmark O 1983 **Competition between perch (*Perca fluviatilis*) and roach (*Rutilus rutilus*) in south Swedish lakes** PhD thesis, Lund University, Lund, Sweden.

[63] Steinarsson A, Imsland AK 2003 Size dependent variation in optimum growth temperature of red abalone (*Haliotis rufescens*). Aquaculture 224, 353–362. (10.1016/S0044-8486(03)00241-2)

[64] Panov VE, McQueen DJ 1998 Effects of temperature on individual growth rate and body size of a freshwater amphipod. Can. J. Zool. 76, 1107–1116. (10.1139/z98-025)

[65] Björnsson B, Steinarsson A, Árnason T 2007 Growth model for Atlantic cod (*Gadus morhua*): effects of temperature and body weight on growth rate. Aquaculture 271, 216–226. (10.1016/j.aquaculture.2007.06.026)

[66] Angiletta MJ, Steury TD, Sears MW 2004 Temperature, growth rate, and body size in ectotherms: fitting pieces of a life-history puzzle. Integr. Comp. Biol. 44, 498–509. (10.1093/icb/44.6.498) 21676736

[67] van Rijn I, Buba Y, DeLong J, Kiflawi M. 2017 Large but uneven reduction in fish size across species in relation to changing sea temperatures. Glob. Change Biol. 23, 3667–3674. (10.1111/gcb.13688) 28296022

[68] Genner MJ et al. 2010 Body size-dependent responses of a marine fish assemblage to climate change and fishing over a century-long scale. Glob. Change Biol. 16, 517–527. (10.1111/j.1365-2486.2009.02027.x)

[69] Arranz I et al. 2016 Density-dependent effects as key drivers of intraspecific size structure of six abundant fish species in lakes across Europe. Can. J. Fish. Aquat. Sci. 73, 519–534. (10.1139/cjfas-2014-0508)

[70] Jeppesen E et al 2010 Impacts of climate warming on lake fish community. Hydrobiology 646, 73–90. (10.1007/s10750-010-0171-5)

[71] Audzijonyte A, Richards SA, Stuart-Smith RD, Pecl G, Edgar GJ, Barrett NS, Payne N, Blanchard JL 2020 Fish body size change with temperature but not all species shrink with warming. Nat. Ecol. Evol. 4, 809–814. (10.1038/s41559-020-1171-0) 32251381

[72] O'Connor MI, Bruno JF, Gaines SD, Halpern BS, Lester SE, Kinlan BP, Weiss JM 2007 Temperature control of larval dispersal and the implications for marine ecology, evolution and conservation. Proc. Natl Acad. Sci. USA 104, 1266–1271. (10.1073/pnas.0603422104) 17213327 PMC1764863

[73] Huss M, Byström P, Strand Å, Eriksson L-O, Persson L 2008 Influence of growth history on the accumulation of energy reserves and winter mortality in young fish. Can. J. Fish. Aquat. Sci. 65, 2149–2156. (10.1139/F08-115)

[74] van de Wolfshaar KE, de Roos AM, Persson L. 2008 Population feedback after successful invasion leads to ecological suicide in seasonal environments. Ecology 89, 259–268. (10.1890/06-2058.1) 18376567

[75] Ohlberger J 2013 Climate warming and ectotherm body size: from individual physiology to community ecology. Funct. Ecol. 27, 991–1001. (10.1111/1365-2435.12098)

[76] Peck LS, Clark MS, Morley SA, Massey A, Rossetti H 2009 Animal temperature limits and ecological relevance: effects of size, activity and rates of change. Funct. Ecol. 23, 248–256. (10.1111/j.1365-2435.2008.01537.x)

[77] Rall BC, Vucic-Pestic O, Ehnes RB, Emmerson MC, Brose U 2010 Temperature, predator-prey interaction strength and population stability. Glob. Change Biol. 16, 2145–2157. (10.1111/j.1365-2486.2009.02124.x)

[78] Becker CD, Genoway RG 1979 Evaluation of the critical thermal maximum for determining thermal tolerance of freshwater-fish. Env. Biol. Fishes 4, 245–256. (10.1007/BF00005481)

[79] Sandström O, Neumann E, Thoresson G 1995 Effects of temperature on life history variables in perch. J. Fish Biol. 47, 652–670. (10.1111/j.1095-8649.1995.tb01932.x)

[80] Huss M, de Roos AM, van Leeuwen A, Casini M, Gårdmark A 2013 Cohort dynamics give rise to alternative stable community states. Am. Nat. 182, 374–392. (10.1086/671327) 23933727

[81] van Leeuwen A, Huss M, Gårdmark A, Casini M, Vitale F, Hjelm J, Persson L, de Roos AM 2013 Predators with multiple ontogenetic niche shifts have limited potential for population growth and top-down control of their prey. Am. Nat. 182, 53–66. (10.1086/670614) 23778226

[82] Persson L, Amundsen P-A, de Roos AM, Klemetsen A, Knudsen R, Primicerio R. 2007 Culling prey promotes predator recovery: alternative states in a whole lake experiment. Science 316, 1743–1746. (10.1126/science.1141412) 17588929

[83] Gilman SE, Urban MC, Tewksbury J, Gilchrist GW, Holt RD 2010 A framework for community interactions under climate change. Trends Ecol. Evol. 25, 325–331. (10.1016/j.tree.2010.03.002) 20392517

[84] Harley CDG 2011 Climate change, keystone predation and biodiversity loss. Science 334, 1124–1127. (10.1126/science.1210199) 22116885

[85] Uszko W, Diehl S, Englund G, Amarasekare P 2017 Effects of warming on predator-prey interactions: a resource-based approach and a theoretical synthesis. Ecol. Lett. 20, 513–523. (10.1111/ele.12755) 28266168

[86] Bates AE, Stuart-Smith RD, Barrett NS, Edgar GJ 2017 Biological interactions both facilitate and resist climate-related functional change in temperate reef communities. Proc. R. Soc. B 284, 20170484 (10.1098/rspb.2017.0484) PMC5474073 28592671

[87] Greig HS, Kratina P, Thompson PL, Palen WJ, Richardson JS, Shurin JB 2012 Warming, eutrophication, and predator loss amplify subsidies between aquatic and terrestrial ecosystems. Glob. Change Biol. 18, 504–514. (10.1111/j.1365-2486.2011.02540.x)

[88] Marino NAC, Romero GQ, Farjalla VF 2018 Geographical and experimental contexts modulate the effect of warming on top-down control: a meta-analysis. Ecol. Lett. 21, 455–466. (10.1111/ele.12913) 29368449

[89] Kratina P, Greig HS, Thompson PL, Carvalho-Pereira TS.A, Shurin JB 2012 Warming modifies trophic cascades and eutrophication in experimental freshwater communities. Ecology 93, 1421–1430. (10.1890/11-1595.1) 22834382

[90] Werner EE, Gilliam JF 1984 The ontogenetic niche and species interactions in size structured populations. Annu. Rev. Ecol. Syst. 15, 393–426. (10.1146/annurev.es.15.110184.002141)

[91] Svensson F, Karlsson E, Gårdmark A, Olsson J, Adill A, Zie J, Snoeijs P, Eklöf JS 2017 *In situ* warming strengthens trophic cascades in a coastal food web. Oikos 126, 1150–1161. (10.1111/oik.03773)

[92] Sorte CJ, Williams SL, Carlton JT 2010 Marine range shifts and species introductions: comparative spread rates and community impacts. Glob. Ecol. Biogeogr. 19, 303–316. (10.1111/j.1466-8238.2009.00519.x)

