# Supplementary material for: Individual variation and interactions explain food web responses to global warming
Source: Philos Trans R Soc Lond B Biol Sci. 2020 Nov 2;375(1814):20190449. doi: 10.1098/rstb.2019.0449 (PMC7662199; doi:10.1098/rstb.2019.0449)
Supplement: Methods and data for figure 2 [file rstb20190449supp1.pdf]

Supplementary material

Individual variation and interactions explain food web responses to global warming

Anna Gårdmark*[1] and Magnus Huss[2]

[1]*Department of Aquatic resources, Swedish University of Agricultural Sciences, Skolgatan 6, SE-742 42 Öregrund, Sweden, ORCID 0000-0003-1803-0622*

[2]*Department of Aquatic resources, Swedish University of Agricultural Sciences, Skolgatan 6, SE-742 42 Öregrund, Sweden, ORCID 0000-0002-5131-6000*

* Author for correspondance (anna.gardmark@slu.se)

Methods

Relative consumption and metabolic rate

Data on consumption rate of European perch (*Perca fluviatilis*) was taken from the experiments by Lessmark (1983), who measured the rate of consumption as percent per day for perch ranging 2-77g at six different temperatures (4, 8, 12, 16, 20, 24 degrees C). Data was extracted from figure 3 page 25 in Lessmark (1983) using a WebPlotDigitilizer 4.2 (https://apps.automeris.io/wpd/). Data was selected for two size groups, 2-6 g and 45-77 g, and converted to relative rates by dividing them by their respective consumption rate at 20 degrees C (which we use as reference temperature) and plotted in Fig. 2A. The relative consumption rates by mass are presented in table S1.

Table S1. Consumption rate of perch at different temperatures relative to consumption rate at 20 degrees C, based on data extracted from a graphical presentation by Lessmark (1983) who measured consumption (%/day) of perch of different weight at different temperatures.

**Size group (g)**	**Weight (g)**	**Temperature (°C)**	**Relative consumption rate**
3 (2-6)	4.72127402	4	0.081016594
3 (2-6)	4.496294994	8	0.192974622
3 (2-6)	3.84780781	12	0.297579408
3 (2-6)	6.113653076	16	0.53875243
3 (2-6)	3.486208786	20	1
3 (2-6)	2.240378278	24	1.771030975
50 (45-77)	76.37235554	4	0.054560309
50 (45-77)	74.25448717	8	0.155668616
50 (45-77)	67.02228364	12	0.209828114
50 (45-77)	57.30481169	16	0.393554861
50 (45-77)	45.44838739	20	1
50 (45-77)	76.9754247	24	1.102909700

To these relative consumption rates we visually fitted a hump-shaped temperature dependence curve that accounts for size-dependent effects of temperature (Gårdmark et al., in prep.); for each size group this corresponds to a separate Sharpe-Schoolfield equation (Schoolfield et al. 1981, Padfield et al. 2016):

$$r(T) = e^{-\frac{E_A}{k}\left(\frac{1}{T}-\frac{1}{T_{ref}}\right)} \cdot (1 + e^{-\frac{E_D}{k}\left(\frac{1}{T}-\frac{1}{T_D}\right)})^{-1} \cdot \left(1 + e^{-\frac{E_D}{k}\left(\frac{1}{T_{ref}}-\frac{1}{T_D}\right)}\right) \quad \text{(S1)}$$

For the relative consumption rates for each of the perch size classes illustrated in Fig. 2A, parameters used correspond to $E_A = 3, E_D = 4, T_{ref} = 293.15K, T_D = 294.7K, k = 8.617332 \cdot 10^{-5}$ in eq. S1 for a 3 g perch, and for the 50 g perch $E_A = 1.95, E_D = 2, T_{ref} = 293.15K, T_D = 295.8K, k = 8.617332 \cdot 10^{-5}$.

As there, to our knowledge, are no studies testing for size-dependent temperature effects in metabolic rate in perch although it is known to occur for many species (Ohlberger et al. 2012, Lindmark et al. 2018), we illustrated this relationship with a size-dependent version of the Arrhenius function scaled to a reference temperature (Gårdmark et al., in prep.):

$$r(T) = m^{c_A(T-T_{ref})} \cdot e^{-\frac{E_A}{k}\left(\frac{1}{T}-\frac{1}{T_{ref}}\right)} \qquad \text{(S2)}$$

To illustrate the corresponding metabolic rate of a 3g and 50 g perch in relation to their metabolic rate at the reference temperature 20 degrees C, we used $E_A = 0.62, c_A = -0.01, T_{ref} = 293.15K, k = 8.617332 \cdot 10^{-5}$ in eq. S2.

The temperature-dependent relative rates of consumption and metabolism for small and large perch are illustrated in Fig. 2A.

Body growth & length-at-age in whole-ecosystem warming experiment

The whole-ecosystem warming experiment, and the individual fish data collected therein, has been reported by Huss et al. (2019), but the description of the experimental set-up and the collection of the data used for Fig. 2B-D are repeated below for clarity.

The experimental site consists of an artificially heated 1 km^2 enclosed coastal ecosystem in the Baltic Sea archipelago called the Biotest Lake. This enclosure was completed in 1977 and built to receive the heated cooling water from the nearby nuclear power plant in Forsmark, Sweden, in which the first reactor was started in 1980. Monitoring of the effects of this artificial heating on the ecosystem was established from 1977 as a paired design, with a reference area in the surrounding archipelago. Since 1980, the water temperature in the heated ecosystem (Biotest Lake) has been ~ 5-10 ˚C above that of the neighbouring reference area.

During the whole study period in Huss et al. (2019) and for the years illustrated in Fig. 2B-D, the Biotest Lake was closed for fish migration (at least for fish > 10 cm, such as adult perch) from the surrounding sea by a grid (removed in 2004) at the outlet, and a strong water current

(80-ca100 m3/s) through the grid that prevented immigration of small fish. This suggests that the two perch populations in the heated ecosystem and in the reference area were separated during the studied years. Fish data from the two areas have been continuously collected since 1977 as part of a monitoring program studying the impact of the nuclear power plant on the natural environment. Fishing has been performed using multi-mesh gillnets, and the data presented by Huss et al. (2019) includes catch per unit effort of perch (CPUE, number of fish per net & night), length-at-age of perch when caught and back-calculated individual somatic growth of perch derived from measured annuli in their operculum bone (see Huss et al. 2019, for details).

In Fig. 2B we illustrate the size-specific body growth over time in the heated ecosystem, before and after heating begun, for two size-classes of perch: 5 mm (corresponding to size at birth) and 140-160 mm (corresponding to the mean length of 3-year-olds), redrawn based on data in Fig. 5a-b in Huss et al. (2019). We also illustrate the length-specific body growth of 5 mm and 170 mm perch from different lake populations, redrawn from the supplement of Van Dorst et al (2019). These lengths corresponds to size of newborn perch and adult size across the lakes with different temperature, respectively (Van Dorst et al. 2019).

In Fig. 2C we illustrate the corresponding changes in length-at-age of 1-year-old and 3-year-old perch in the heated ecosystem for the same periods, redrawn based on data in Fig. 4a-b in Huss et al. (2019). Observations of length of 1-year-old perch in lakes of different mean temperatures illustrated in Fig. 2C are taken from Van Dorst et al. (2019), whereas length of 5-year-old perch were derived from the same data on perch (unpublished data; Van Dorst, R., personal communication).

Size distribution, mean length and skewness in length

Mean length and skewness in length distribution (higher values meaning more small relative to large individuals) of lake perch populations across temperature plotted in Fig. 2D (black dots in bottom panels) were redrawn from Van Dorst et al. (2019). For the perch populations in the whole-ecosystem warming experiment (top panels in Fig. 2D and coloured symbols in bottom panels), we calculated the length distribution of perch in the heated ecosystem using

catch per unit effort data from the same monitoring programme as used by Huss et al. (2019). Fishing is done with two 35 m long and 3 m deep linked stationary multi-mesh nets (each with five different mesh sizes: 17, 22, 25, 33, and 50 mm) that are deployed over night (see Olsson et al. 2012) at each net station. For these analyses we used catches from August shortly after warming started (1984; week 33 in the heated ecosystem and week 31 in the reference area) and several generations later (2003; week 35 in the heated ecosystem and week 33 in the reference area). We used only catches from the first night of fishing during each of these periods, as catches decline over subsequent nights. Catches from five net stations in the heated ecosystem and four net stations in the reference area (of similar depth as in the heated ecosystem) were used for the calculations. In the beginning of the monitoring programme, fish were measured in 5-cm length groups, whereas they later on were measured in 1-cm length groups (yielding different resolution of the length distributions in 1984, Table S2a, and 2003, Table S2b).

Table S2a. Length distribution (from catch per unit effort) of the perch population in the heated coastal ecosystem and in the reference area, respectively, in year 1984.

**Length group** (cm)	**Heated ecosystem** (number of individuals net^{-1} $night^{-1}$)	**Reference area** (number of individuals net^{-1} $night^{-1}$)
5-10	0	29.5
10-15	15	16.5
15-20	3.8	5.25
20-25	0	0.5
25-30	0.4	0.5
30-35	0	0

Table S2b. Length distribution (from catch per unit effort) of the perch population in the heated coastal ecosystem and in the reference area, respectively, in year 2003. (Table continues on next page)

**Length group** (cm)	**Heated ecosystem** (number of individuals net^{-1} $night^{-1}$)	**Reference area** (number of individuals net^{-1} $night^{-1}$)
8	0	0
9	0	0
10	0	0
11	0	0.25
12	0.6	3.25
13	0.2	5.5
14	0	1
15	0	1.5
16	1	3.75
17	2.6	3.25
18	2.2	0.5
19	5.4	1.25
20	5	1
21	4.8	0.75
22	0.6	0
23	0.6	0.5
24	1.4	0.5
25	0.6	0
26	1	0.25
27	1	0
28	0.4	0
29	0.6	0
30	0.4	0
31	0	0.25
32	0.2	0
33	0.4	0
34	0	0

**Length group** (cm)	**Heated ecosystem** (number of individuals net^{-1} night^{-1})	**Reference area** (number of individuals net^{-1} night^{-1})
30	0.4	0
31	0	0.25
32	0.2	0
33	0.4	0
34	0	0
35	0.2	0
36	0.8	0.25
37	0	0
38	0	0.25
39	0.2	0
40	0	0
41	0	0
42	0	0
43	0.2	0
44	0.2	0
45	0	0
46	0.4	0
47	0	0
48	0.2	0

Based on these data, i.e., the numbers of individuals per length caught per net and night (Fig. 2D, Table S2a-b), we calculated the mean length of perch in the heated ecosystem and in the reference area for these two years as well as the skew in the length distribution (coloured dots in Fig. 2D lower panels, Table S3; black dots are lake perch populations directly redrawn from Van Dorst et al. 2019). Because the smallest perch individuals are not representatively caught in these mesh sizes, they were removed from the calculation of the means and skewness (<10 cm 1984 due to the low number of size classes measured, <16 cm 2003). Temperature in Fig. 2D (lower panels, Table S3) is the mean temperature (1 m from surface in 2003, and bottom depth (ca 4 m) in 1984) during the first fishing night. Temperature was

measured when deploying and when retrieving the nets at the centre of each set of net stations, in the heated ecosystem and in the reference area, respectively.

Table S3. Mean length and skewness in length distributions of perch in the population in the heated ecosystem and in the reference area, respectively in 1984 and 2003, and corresponding temperatures during those days of fishing. A high skewness corresponds to a large proportion of small compared to large individuals.

**Year**	**Ecosystem**	**Temperature**	**Mean length (cm)**	**Skewness in length**
1984	Heated	22.2	13.8	3.00
1984	Reference	19.1	14.2	2.33
2003	Heated	23.8	22.4	2.22
2003	Reference	19.0	19.2	2.78

Population biomass production, population biomass and prey biomass

Production of biomass (Fig. 2E) as well as standing stock biomass (Fig. 2F) of perch populations in lakes of different temperatures were redrawn directly from Van Dorst et al (2019). Van Dorst et al. (2019) used catch per unit effort (gram perch per m^2 net area and night deployed) as a standardized measure across lakes for population biomass. Biomass production (in gram perch per m^2 net area and year) in Van Dorst et al. (2019) was calculated for each lake as length-specific body growth (in biomass) multiplied by the total biomass (catch per unit effort) of perch per centimetre length-class, summed across the length classes in the lake. By presenting the production per area of the nets used when sampling perch size-distributions, the estimated biomass production becomes comparable between lakes.

Fig. 2F is a conceptual illustration of prey biomass responses, and not based on data.

Supplementary references

Huss, M,. Lindmark, M., van Dorst, R.M., Jacobson, P. & A. Gårdmark. 2019. Large-scale experimental evidence of gradual size-dependent shifts in body size and growth of fish in response to warming. *Global Change Biol* **25**, 2285-2295. (doi: 10.1111/gcb.14637)

Olsson, J., Bergström, L. & Gårdmark, A. 2012. Abiotic drivers of coastal fish community change during four decades in the Baltic Sea. *ICES J Mar Sci* **69**, 961-970.

Padfield, D., Yvon-Durocher, G., Buckling, A., Jennings, S. & Yvon-Durocher, G. 2016. Rapid evolution of metabolic traits explains thermal adaptation in phytoplankton. *Ecol Letters* **19**, 133-142.

Schoolfield, R.M., Sharpe, P.J.H. & Magnuson, C.E. 1981. Non-linear regression of biological temperature-dependent rate models based on absolute reaction-rate theory. *J Theor Biol* **88**, 719-731.

Van Dorst, R.M., Gårdmark, A., Svanbäck, R., Beier, U., Weyhenmeyer, G.A. & M. Huss. 2019. Warmer and browner waters decrease fish biomass production. *Global Change Biol* **25**, 1395-1408. (doi: 10.1111/gcb.14551)
